# Supplementary material for: Simultaneous observation of free and defect-bound excitons in CH3NH3PbI3 using four-wave mixing spectroscopy
Source: Sci Rep. 2016 Dec 15;6:39139. doi: 10.1038/srep39139 (PMC5156914; doi:10.1038/srep39139)
Supplement: Supporting Information [file srep39139-s1.pdf]

Supplementary Information for "Simultaneous observation of free and defect-bound excitons in $CH_3NH_3PbI_3$ using four-wave mixing spectroscopy"

Samuel A. March, Charlotte Clegg, Drew B. Riley, Daniel Webber, Ian G. Hill, and Kimberley C. Hall*

Department of Physics and Atmospheric Science, Dalhousie University, Halifax, Nova Scotia B3H4R2, Canada

E-mail: kimberley.hall@dal.ca

Phone: (902) 494 7109. Fax: (902) 494 5191

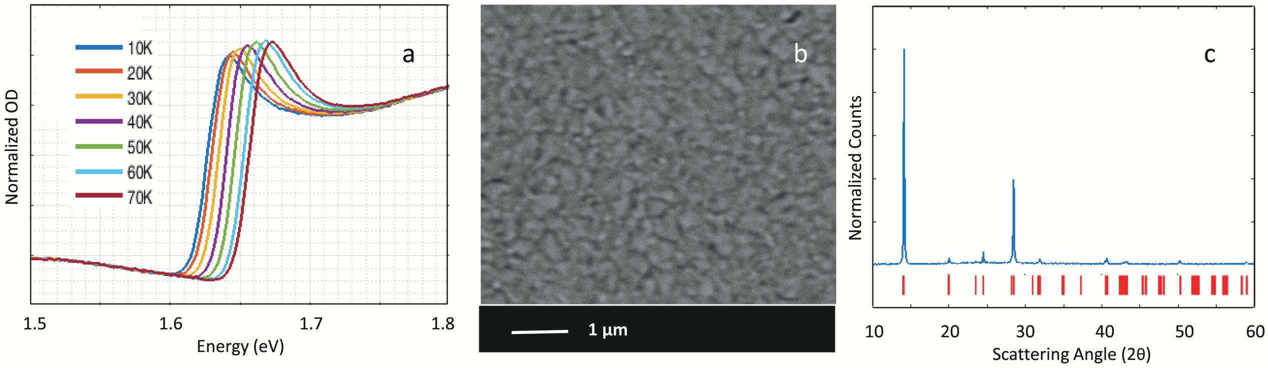

Figure S1: Characterization of the $CH_3NH_3PbI_3$ thin film, showing **a** Temperature-dependent linear absorption, **b** scanning electron microscopy, and **c** x-ray diffraction. **b** and **c** were taken at room temperature. The red lines in **c** indicate the calculated peak positions for the tetragonal phase.

*To whom correspondence should be addressed

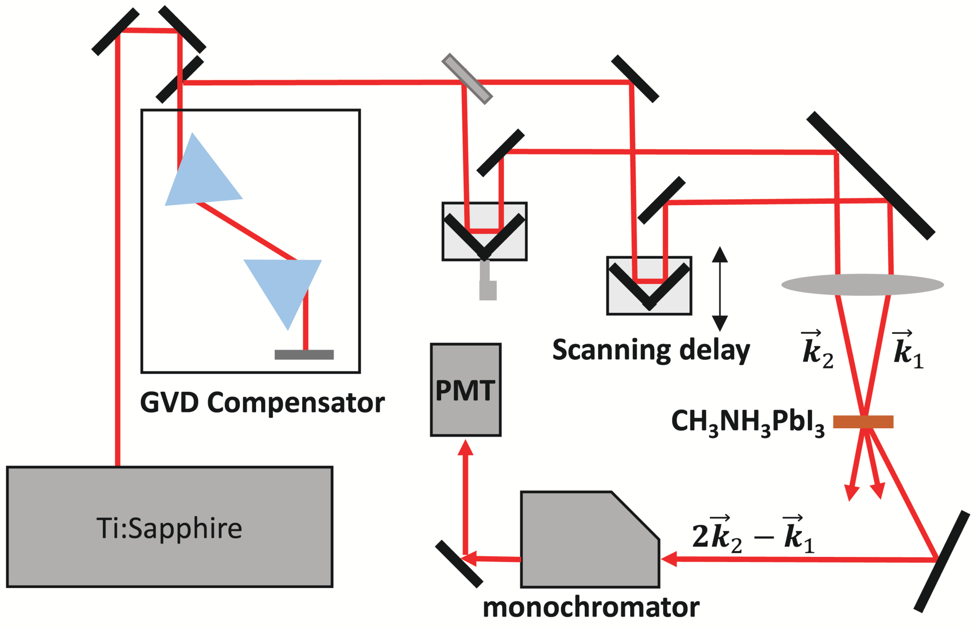

Figure S2: Schematic diagram of the two-pulse degenerate four-wave mixing apparatus.

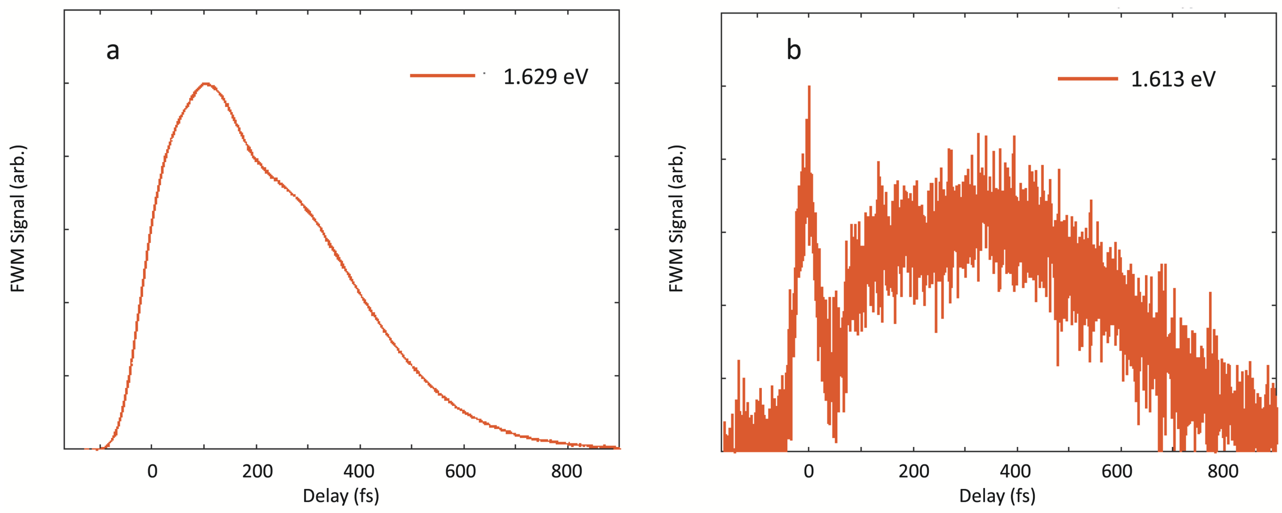

Figure S3: Dependence of FWM signal on time delay for detection resonant with the defect-bound exciton (**a**) and free exciton (**b**). Although the T_2 time is not well defined due to the nonexponential dependence, a rough estimate of the duration of the coherence signal ~0.5 ps corresponds to a homogeneous linewidth of 2.7 meV, approximately 5 times smaller than the measured linewidth indicating inhomogeneous broadening.
